# COVID-19 Vaccines and the Efficacy of Currently Available Vaccines Against COVID-19 Variants

**DOI:** 10.7759/cureus.24927

**Published:** 2022-05-11

**Authors:** Suganya Panneer Selvam, Pratibha Ramani, Ramya R, Sandhya Sundar, Lakshmi T A

**Affiliations:** 1 Oral Pathology, Saveetha Dental College & Hospital, Chennai, IND; 2 Oral Biology, Saveetha Dental College & Hospital, Chennai, IND

**Keywords:** covaxin, booster dose, efficacy, moderna, vaccine, omicron, corona

## Abstract

Severe acute respiratory syndrome coronavirus 2 is the seventh member of the Coronaviridiae family of viruses, which are thought to be transmitted by Chinese horseshoe bats. The virus undergoes mutations leading to variants such as B.1.1.7 (alpha), B.1.351 (beta), P.1 (gamma), and B.1.617 (delta), as well as the recent variant B.1.1.529 (omicron), which has around 30 deletions, making it a severely mutated form that lowers vaccination-induced protection. Vaccine efficacy is usually expressed as relative risk reduction, which is based on the ratio of attack rates with and without a vaccine, whereas absolute risk reduction is based on the entire population. Rather than two doses, recent research suggests that a third dose/booster dose may aid in protection against future variants. The constant influx of mutant variations is putting a strain on vaccine production. Despite the challenges, we are optimistic that the epidemic will be eradicated by achieving mass immunity and by ensuring that everyone receives vaccines at a faster rate.

## Introduction and background

Coronaviruses belong to the Coronaviridiae family in the Nidovirales order. Bats were considered to be the only reservoir, and the first animal to be documented was the Chinese horseshoe bats [1-3]. The human-to-human transmission of the virus occurs due to close contact with infected persons, and aerosols generated during coughing or sneezing can penetrate the human body during inhalation through the nose or mouth [4]. The earliest case was documented in December 2019, and it is the seventh member of the coronavirus family to infect humans [5]. The International Committee on Taxonomy of Viruses (ICTV) named the virus severe acute respiratory syndrome coronavirus 2 (SARS-CoV-2) and the disease coronavirus disease 2019 (COVID-19). The higher transmission rate of SARS-CoV-2 could be a genetic recombination event at the S protein in the RBD region [4]. The virus undergoes mutations resulting in variants such as B.1.1.7 (alpha), B.1.351 (beta), P.1 (gamma), B.1.617 (delta), and the newer variant omicron (B.1.1.529). Omicron has more than 30 deletions and is considered to be a heavily mutant variant that reduces vaccine-mediated immunity [6]. Vaccines are the only tool to control this pandemic outbreak as no specific therapies have been introduced to date. Numerous vaccines have been manufactured, and this article provides information about COVID-19 vaccines and their efficacy against coronavirus variants.

## Review

Structure of COVID-19

Coronavirus is an enveloped virus approximately 120 nm in diameter with a single-stranded RNA genome. The RNA genome consists of a 5’ methyl-guanosine cap, poly (A) tail, and 29,903 nucleotides. It consists of an open reading frame (ORF) encoding non-structural proteins for replication, four structural proteins (spike, envelope, membrane, nucleocapsid), and numerous accessory proteins. The spike glycoprotein binds to angiotensin-converting enzyme 2 (ACE2) in humans for cell entry. The spike protein is cleaved by host proteases into S1 and S2 subunits where S1 is responsible for receptor recognition and S2 for membrane fusion. S1 can be divided into the C-terminal domain and N-terminal domain, with the C-terminal domain showing a stronger affinity for human ACE2. After attachment, the conformational change in the S protein facilitates the fusion of the virus envelope with the cell membrane. The viral RNA genome is released into the cytoplasm and translated into viral replicase polyproteins pp1a and pp1ab3 which are cleaved into small products by virus-encoded proteinases. The polymerase transcribes a series of subgenomic mRNAs by discontinuous transcription which are finally translated into viral structural proteins (S, E, M, N proteins). The N protein combines with the positive-stranded genomic RNA to form a nucleoprotein complex. The structural protein and nucleoprotein complex are assembled within the viral envelope and then released from the infected cell [5].

Entry mechanism of coronavirus

The glycoprotein spikes on the outer surface of the virus are responsible for the attachment and entry of the virus into host cells. The receptor-binding domain (RBD) is loosely attached among viruses and this is how it infects multiple hosts. Coronavirus depends on cellular proteases such as human airway trypsin-like protease (HAT), cathepsins, and transmembrane protease serine 2 that split the spike protein and help in further penetration. Research on animal studies has revealed that mutation at spike glycoprotein is responsible for severe infection and that therapeutic agents must target spike glycoproteins

COVID-19 vaccines

Broad-spectrum antibiotics and antiviral drugs were initially used; however, remdesivir has shown promising results. The blood plasma from recovered patients has been injected into infected patients and has shown rapid recovery. Monoclonal antibodies (CR3022) had been found to bind with the spike RBD of SARS-CoV-2 without overlapping with the ACE2 receptor-binding motif. The major hurdle in creating a vaccine is to overcome cross-resistance. Many companies such as Moderna Therapeutics, Inovio Pharmaceuticals, Novavax, Vir Biotechnology, Ster Mirna Therapeutics, Johnson & Johnson, VIDO-Intervac, GeoVax-BravoVax, Clover Biopharmaceuticals, Curevac, and Codagenix had been working for the development of vaccines against COVID-19 [4].

RNA/DNA Vaccines

It uses the host cell’s transcription and translation processes to express the vaccine antigen that is encoded by the injected nucleotide. COVID-19 RNA-based vaccines are delivered in lipids, and the production is a very quick process [7]. However, freezing huge amounts of the vaccine is challenging [8]. The mRNA-1273 vaccine administered in lipid particles that encode for spike protein triggered a good antibody response [9].

Viral Vector-Based Vaccines

The genetic sequence of the foreign viral antigen inserted into the genome is the baseline of viral vector vaccines. This antigen is either secreted inducing B-cell/antibody response or digested within the target cells inducing a potent CD8+T cell response [10]. However, the vaccine could be neutralized if the vaccinated individuals have pre-existing antibodies against the vector virus. The recent viral vector vaccine against COVID-19 is mostly adenovirus-based. Antibody and T-cell responses were increased on day 28 after vaccination even after its reduction with the presence of pre-existing anti-adenovirus antibodies [11]. ChAdOx1, also known as AZD1222 (Covishield), has increased antibody titers after a booster dose at 28 days [12].

Protein-Based Vaccines

Protein-based vaccines/inactivated vaccines with aluminum salt adjuvants are currently used in routine childhood immunization. One such vaccine for COVID-19 is PiCoVacc, also called CoronaVac, for which clinical trials are ongoing in healthy individuals and the elderly population [13,14]. β-propiolactone inactivated SARS-CoV-2WIV04 is another alum-adjuvanted strain with more antibody titers, but these types of vaccines have a chance of eliciting antibody enhancement of disease [15,16]. The other adjuvant vaccines in the trial are the adjuvanted S-trimer vaccine (SCB-19) and subunit vaccine NVX-CoV2373 adjuvanted with matrix-M1 saponins [17].

Vaccines Based on Accentuated SARS-CoV-2 Viruses

This is the most traditional technique where the virus has been weakened so that it will not cause the disease. Though it is effective in strengthening the immune system and inducing a strong immune memory, none of the vaccines for COVID-19 have reached the stage of clinical trials [18].

Vaccines Based on Inactivated SARS-CoV-2 Viruses

This vaccine is based on killed viruses but has a short immune memory. This is not only designed to direct against the spike protein but also many other SARS-CoV-2 antigens. CoronaVac and Sinopharm from China and Covaxin from India were successfully administered to the population after completing phase III trials [18].

Vaccines Based on SARS-CoV-2 Proteins

The vaccines are based on the proteins present on the surface of microbes that are produced in vitro with the help of DNA recombinant technology. The targets of these vaccines are the spike protein and, to some extent, nucleoprotein. Novavax from the United States and Co-VLP have completed phase III trials and have been administered to the population in two doses [18].

Naked DNA-Based Vaccines

DNA plasmids enter human cells after vaccination and induce cells to produce the target protein for a while, thus stimulating the production of antibodies and activation of killer T-cells. Although DNA vaccines are used in the veterinary field, none of them have been administered to humans. However, Inovio from the United States and Genexine from Korea are under trial [18].

mRNA-Based Vaccines

mRNA vaccines induce the cell to produce antigen proteins coded by the mRNA where it is carried by liposomes as nanoparticles, unlike DNA-based vaccines. Pfizer from the United States, Moderna from the United States, Arcturus Ther from Singapore, and CureVac from Germany have been administered to the population after passing clinical trials successfully [18]. In addition, an inhaled form of vaccine from the University of Oxford, UK, has not yet reached phase III trial.

Vaccines Based on Viral Vectors

The DNA coding for the spike protein is inserted into the virus as the virus has the ability to induce immunity by delivering mRNA into the cells. Sputnik from Russia, Covishield from India, Johnson & Johnson from the United States, and Ad5-nCoV use viral vectors to target spike protein [8].

Efficacy of COVID-19 vaccines

Vaccine efficacy is generally reported as relative risk reduction (RRR) which uses the relative risk (RR) that uses the ratio of attack rates with or without a vaccine, whereas absolute risk reduction (ARR) uses the whole population. However, ARR tends to be ignored as it gives much less effect than RRR. Efficacy provides the RRR of 95% for the Pfizer-BioNTech, 94% for Moderna-NIH, 91% for Gamaleya, 67% for Johnson & Johnson, and 67% for AstraZeneca Oxford vaccines. However, the spectrum entirely changes when the effectiveness of a vaccine is calculated in terms of the number needed to vaccinate (NNV) to prevent one more case of COVID-19, with 81 for Moderna-NIH, 78 for AstraZeneca Oxford, 108 for Gamaleya, 84 for Johnson & Johnson, and 119 for Pfizer-BioNTech vaccines [19].

BNT162b2/Pfizer/BioNTech was the first vaccine approved by the European Medicines Agency against COVID-19 which is administered intramuscularly after dilution in a cycle of two doses 21 days apart [20,21]. Preclinical data include the investigation of immunogenicity and antiviral properties in mice and non-human primates, which proved that the BNT162b2 vaccine protected the lungs of immunized rhesus macaques from COVID-19 [22]. In a phase I/II study, where BNT162b2 was administered to adults, the geometric mean concentrations of RBD-binding immunoglobulin G (IgG) were high after 21 days of the first dose (534-1778 U/mL) when compared to the human convalescent serum (HCS 602 U/mL) after 14 days of the infection confirmed by polymerase chain reaction (PCR). This vaccine showed 95% efficacy and proved that early protection occurs 12 days after the first dose. Another study was performed to evaluate the humoral and adaptive immune response for BNT162b1 from phase I/II studies, in which GMC of anti-RBD antibodies were more in vaccinated individuals (3,920-18,289 U/mL) when compared to HCS (602 U/mL) [23,24]. Further, the efficacy of the vaccine against variants was also tested which shows high neutralizing capacity in all variants, with increased CD4+ and CD8+ RBD-specific T-lymphocytes [25]. However, BNT162b2 was recommended as it is associated with lower incidence and severity of systemic complications when compared to BNT162b1. A two-dose regimen of BNT162b2 conferred 95% efficacy against COVID-19 in a study conducted among 43,448 participants who were injected with the COVID-19 vaccine and saline placebo. Though the efficacy after the first dose was only 51%, it gradually increased to 91% within the first seven days of the second dose [26]. The vaccine efficacy among participants with hypertension also yielded an efficacy of 94.6%, consistent with other participants [27]. Another study among healthcare workers in Israel to evaluate the efficacy was found to be 30% in 1-14 days and 75% in 15-28 days [27-29]. In another study, the efficacy in preventing hospitalization after severe infection was found to be 80%, and a single dose helped in preventing death by 85% [30-32].

The Sputnik V vaccine is a vector vaccine based on adenovirus DNA administered over a course of two days, initially produced in Russia. In the previous study, the efficacy of the Sputnik vaccine with two vector components, namely, recombinant adenovirus type 26 (rAd26) and adenovirus type 5 (rAd5), manufactured in two formulations - frozen (0.5 mL) and lyophilized (1 mL of sterile water per dose) was demonstrated. There was increased production of CD4+ and CD8+ after administration of the frozen form than of the lyophilized form, and there was also increased production of interferon-gamma (IFN-γ) in all vaccinated individuals. The titer of neutralizing antibodies matches the convalescent human serum of those who recovered from recent infection [33]. The phase 3 trial of the Sputnik vaccine gives an efficacy of 91.6% (confidence interval (CI) = 95%), and, interestingly, it was 91.8% for older adults above 60 years. However, initially, the efficacy was 73.6% till 21 days after the first dose [33,34]. In another study where the vaccine was administered in older individuals after the first dose, the efficacy was found to be 78.6% of laboratory-confirmed SARS-CoV-2 infections, 94% in hospitalization, and 93% in preventing death among them, thus delaying the second dose can be followed to increase neutralizing antibodies [35]. The vaccine resists the variants of the COVID-19 virus with a 6.1-fold reduction in the neutralizing activity [36].

Covishield/AZD1222/ChAdOx1 is a viral vector intramuscular vaccine manufactured by the Serum Institute of India and is administered in two doses. In a study among 52 healthy individuals who received the second dose after 56 days of the first dose, there were no systemic reactions such as pyrexia as in the first dose [37-39]. After the first dose, discrete populations of T-cells, B-cells, and natural killer (NK) cells were detected. There was no sex difference and association between the age and magnitude response in this study among individuals aged from 18 to 55 years. The IgG spike was similar to that of plasma from convalescent patients with SARS-CoV-2. The CD4+ and CD8+ secrete antigen-specific cytokines with CD8+ T-cells expressing CD107a, which is a degranulation marker, indicating its cytotoxic effect after vaccination. However, the predominant cytokines were IFN-γ and CD8+ cells [38]. The levels of anti-spike IgG were found to be lower when the individuals received half-booster doses; however, it was increased to 10-fold when they received prime booster doses [38,39]. The total antigen-specific T-cell response was increased after 14 days of vaccination [37]. The second dose either given after 28 days or 56 days after the first shot provides a similar response in producing the antibodies [39]. Preclinical studies in Rhesus macaques prevent SARS-CoV-2-mediated pneumonia [40]. After administration of Covishield with booster dose in older adults, microneutralization assay peaked by day 42, the antibody titers were not raised after the booster dose in all age groups, and the immunization response in older adults was very similar to young adults. The anti-spike IgG levels correlate with neutralizing antibody titers for all age groups (18-55, 56-69, 70, and older) [41]. Another study with 23,848 participants across different parts of the world such as the UK, Brazil, and South Africa was conducted to estimate the efficacy. Interestingly, those who received two standard doses had lesser efficacy (70.4%) than those who received a low dose (2.2 × 1,010 viral particles) vaccine at the first dose followed by the standard booster dose (5 × 1,010 viral particles) (90%). The second dose within six weeks of the first dose provides less efficacy (53.4%) than the one with more than six weeks between the first and second dose (65.4%). Thus, delaying the second dose helps in increasing the efficacy, as well as overcoming the low availability of vaccines in this pandemic situation [42].

Covaxin/BBV152 is an inactivated virus-based COVID-19 vaccine formulated by Bharat Biotech and administered intramuscularly in two doses similar to any other COVID-19 vaccine. In a study with 375 healthy individuals, the vaccine (3 µg and 6 µg with IMBG) was given randomly to evaluate the immune response and efficacy. The most common adverse effects reported were pain in the injection site (5%), fatigue (3%), and headache (3%). The IgG titers were increased after the second dose, and the efficacy was 87.9% with 3 µg and 91.9% with 6 µg, thus IgG titers do not have any effect on the concentration [43]. However, both the doses elicit CD3+, CD4+, and CD8+ leading to IFN-γ production, and the neutralizing antibodies remained elevated for three months after the second vaccination. In the follow-up study with 380 participants, the neutralizing antibody titers were similar to the convalescent serum, and the same results were obtained in other studies [31,44,45].

The Janssen/Johnson & Johnson is another viral vector-based vaccine that requires only a single dose. The first dose of the Johnson & Johnson vaccine provides an efficacy of 76.7% after 14 days of administration of the first dose. However, a phase 3 trial showed an efficacy of 66.9% after 14 days and 66.1% after 28 days of vaccination. The efficacy of preventing severe infection was 76.7% after 14 days and 85.4% after 28 days which is quite high [46,47]. However, other studies reveal that it was 66% effective in preventing symptomatic COVID-19, 85% in severe COVID-19, and 100% in preventing hospitalization or death [48,49].

COVID-19 vaccines against variants of coronavirus

**Figure 1:**
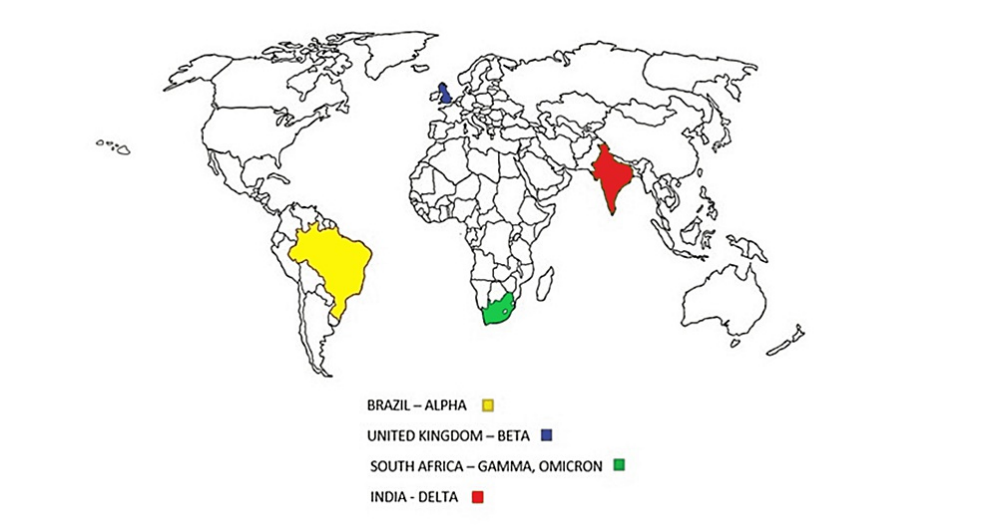
The earliest documented samples of COVID-19 variants according to the World Health Organization. Yellow depicts the alpha variant, blue depicts the beta variant, green depicts the gamma variant, and red indicates the delta variant and their emergence in the respective countries. COVID-19: coronavirus disease 2019

The virus mutations have resulted in variants such as B.1.1.7 (alpha), B.1.351 (beta), P.1 (gamma), B.1.617 (delta), and the newer omicron (B.1.1.7), which has enhanced transmissibility and fatality rates [50-52]. The S genes of B.1.351 and P.1 have various mutations and that might be the reason for re-infection by vanishing neutralizing antibodies formed during infection by the alpha variant [53,54]. The recent delta variant is characterized by spike protein mutations. P681R is at the S1-S2 cleavage site, and this variant has replication at its peak with higher transmissibility [55]. The first case of the recent omicron (B.1.1.529) with mutations in the RBD and NTD first emerged in South Africa in November 2021. The risk of reinfection is 5.4 times greater with omicron than that of the delta variant [56,57]. The emergence of these variants globally is illustrated in Figure 1.

The efficacy of single doses of BNT162b2 and ChAdOx1nCoV-19 vaccines against the delta variant is approximately 36% and 30%, respectively. However, the second dose increases the efficacy to 88% and 67%, respectively. A study from Scotland revealed that the efficacy of Covaxin is 81% against the alpha variant and 61% against the delta variant [58]. The efficacy of Covaxin against the delta variant is around 65% according to a phase 3 trial study [59]. The efficacy of Pfizer and AstraZeneca after the second dose was found to be 78% and 61%, respectively [60]. However, Pfizer-BioTech and Oxford-AstraZeneca vaccines were highly efficacious against delta variants but dropped over time [61]. Alpha variants have a 1.8-2-fold reduction in neutralizing antibodies of Moderna, Sputnik, and Novovax vaccines. The neutralizing capacity for the P.1 variant among vaccinated individuals was reduced by 6.7 for the Pfizer vaccine and 4.5 for the Moderna vaccine [62]. Delta variants have a 7-10 fold reduction in neutralizing antibody levels of Pfizer, Moderna, and Sputnik, and a 2-3-fold reduction in the case of Covishield and Covaxin; however, the sample study with Johnson and Johnson showed that it neutralizes the delta variant similar to Sputnik. However, Sputnik against B.1.351 variant showed a 6.1-fold reduction in neutralizing antibodies [63]. In another study, sera from AstraZeneca recipients showed a 4.1-32.5-fold reduction in neutralizing activity against B.1.351 [64]. However, the reduction in the neutralizing antibodies in the sera of individuals receiving Moderna and BioNTech vaccines was 6.5-8.6-fold [62]. ZyCov-D is another DNA vaccine that is claimed to be effective against the delta variant in clinical trials [65]. The efficacy of these vaccines and the reduction in the neutralizing capacity of COVID-19 vaccines against variants of COVID-19 are presented in Table 1 and Table 2. Vaccinated people affected with the alpha variant had a lower viral load, whereas vaccinated individuals infected with the delta variant had a higher viral load. Thus, irrespective of the vaccine administered, the efficacy is reduced against the delta virus, but still effective in reducing fatality. Until October 2021, globally, 676 crore doses were given and 288 crore individuals were fully vaccinated, that is, 36.9% of the population. In India, 99.7 crore doses were administered to 29.2 crore individuals (21.1%) who are fully vaccinated [66]. The research is still ongoing regarding vaccine efficacy against omicron; however, researchers have found that two doses of the Pfizer vaccine provide 70% infection against hospitalization and 33% against infection [67]. As the efficacy wanes over time, researchers are insisting on having a third dose/booster dose which helps in boosting the efficacy. Recent studies have focused on the combination of booster doses for better efficacy. There was no increase in the efficacy against omicron even after the two doses of AstraZeneca, and this variant reduced the efficacy of Pfizer/Moderna to 10% even after two doses which increased to 40-50% and the effectiveness against hospitalization also increased to 88% after the booster dose [68]. The booster dose increases the neutralizing antibody titers by 25-fold with the Pfizer vaccine [69]. The recent study of vaccine BNT162b2 among the pediatric population (5-11 years) showed 95% efficacy after two doses [70]. Because the efficacy of vaccines against the omicron variant is under investigation, the data available to date is presented in Table 3; however, this might change as studies are published.

**Table 1:** The efficacy of the first and second doses of COVID-19 vaccines against alpha and delta variants. COVID-19: coronavirus disease 2019; CI: confidence interval

Authors	Vaccination doses	Alpha variant (95% CI)	Delta variant (95% CI)	Omicron
Polack et al. [26]	BNT162b2			
	Dose 1	51%	-	No data
	Dose 2	91%	-	No data
Chung et al. [32]	Moderna			
	Dose 1	71%		No data
	Dose 2	91%		No data
	Covaxin			
Sheikh et al. [58]	Dose 2	81%	61%	No data
Ella et al. [59]	Dose 2	-	65%	No data
Lopez Bernal et al. [60]	ChAdOx1 nCoV-19			
	Dose 1	48.7 (45.2–51.9)	30.0 (24.3–35.3)	No data
	Dose 2	74.5 (68.4–79.4)	67.0 (61.3–71.8)	No data
	Pfizer			
	Dose 2	92	78 (90 days)	No data
	AstraZeneca			
	Dose 2	69	61 (90 days)	
Lopez Bernal et al. [60]	BNT162b2			
	Dose 1	47.5 (41.6–52.8)	35.6 (22.7–46.4)	No data
	Dose 2	93.7 (91.6–95.3)	88.0 (85.3–90.1)	No data
Collie et al. [67]	Pfizer			
	Dose 2	-	-	70

**Table 2:** Reduction in the neutralizing capacity of COVID-19 vaccines against alpha, beta, delta, and omicron variants of COVID-19. COVID-19: coronavirus disease 2019; UI: under investigation

COVID-19 vaccines	Alpha	Beta	Delta	Omicron
Covaxin	Unaffected	3	3	UI
Covishield	2.5–9	0–31	2	UI
Pfizer	0–4	1–4	7–10	4–6 lower than wild type
Moderna	0–2	1–28	7	UI
Sputnik	0	7	-	UI
Johnson & Johnson	<1	5–10	-	UI

**Table 3:** Efficacy of COVID-19 vaccines against the omicron variant. Because the efficacy of vaccines against the omicron variant is under investigation, the data available to date has been listed in the table which might change when more studies are published. COVID-19: coronavirus disease 2019

Vaccines	Dose	Efficacy against hospitalization	Efficacy against infection
Pfizer	Dose 2	70%	30–40%
	Booster dose	88%	-
Moderna	Dose 2	78%	-
	Booster dose	88%	-

## Conclusions

Though we are not free from this pandemic yet, vaccines have really helped to overcome the fatality globally. Hence, the main focus now should be on vaccine availability and its distribution globally. More than anything, it should reach everyone equally. It would be appreciable if vaccines are developed for newborns and children. Vaccine production is a lengthy, tedious process, and preparing it during a pandemic leads to a financial crisis and a doubtful immune response. In addition, the mutated variants make the job really challenging more than the population and mass vaccine production. Despite the challenges, we hope that the pandemic ends by conquering mass immunity by administering vaccines to every individual at a faster rate.
